# Predictive Modeling of Factors Influencing Adherence to SGLT-2 Inhibitors in Ambulatory Care: Insights from Prescription Claims Data Analysis

**DOI:** 10.3390/pharmacy12020072

**Published:** 2024-04-22

**Authors:** Nadia Khartabil, Candis M. Morello, Etienne Macedo

**Affiliations:** 1 Center of Graduate Studies, West Coast University, Los Angeles, CA 90004, USA; 2 School of Pharmacy, University of California-San Diego, La Jolla, CA 92093, USA; cmmorello@health.ucsd.edu; 3 School of Medicine, University of California-San Diego, La Jolla, CA 92093, USA; emmacedo@health.ucsd.edu

**Keywords:** adherence, ambulatory care, SGLT-2 inhibitors, diabetes, models, statistical, administrative claims, healthcare

## Abstract

Sodium-glucose cotransporter 2 inhibitors (SGLT2i) are novel oral anti-hyperglycemic drugs that demonstrate cardiovascular and metabolic benefits for patients with type 2 diabetes (T2D), heart failure (HF), and chronic kidney disease (CKD). There is limited knowledge of real-world data to predict adherence to SGLT-2i in an ambulatory setting. The study aims to predict SGLT-2i adherence in patients with T2D and/or HF and/or CKD by building a prediction model using electronic prescription claims data presented within EPIC datasets. This is a retrospective study of 174 adult patients prescribed SGLT-2i at UC San Diego Health ambulatory pharmacies between 1 January 2020 to 30 April 2021. Adherence was measured by the proportion of days covered (PDC). R packages were used to identify regression and non-linear regression predictive models to predict adherence. Age, gender, race/ethnicity, hemoglobin A1c, and insurance plan were included in the model. Diabetes control based on hemoglobin A1c (HbA1c) and the glomerular filtration rate (GFR) was also evaluated using Welch *t*-test with a *p*-value of 0.05. The best predictive model for measuring adherence was the simple decision tree. It had the highest area under the curve (AUC) of 74% and accuracy of 82%. The model accounted for 21 variables with the main node predictors, including glycated hemoglobin, age, gender, and insurance plan payment amount. The adherence rate was inversely proportional to HbA1c and directly proportional to the plan payment amount. As for secondary outcomes, HbA1c values from baseline till 90 days post-treatment duration were consistently higher in the non-compliant group: 7.4% vs. 9.6%, *p* < 0.001 for the PDC ≥ 0.80 and PDC < 0.80, respectively. Baseline eGFR was 55.18 mL/min/1.73m^2^ vs. 54.23 mL/min/m^2^ at 90 days. The mean eGFR at the end of the study (minimum of 90 days of treatment) was statistically different between the groups: 53.1 vs. 59.6 mL/min/1.73 m^2^, *p* < 0.001 for the PDC ≥ 0.80 and PDC < 0.80, respectively. Adherence predictive models will help clinicians to tailor regimens based on non-adherence risk scores.

## 1. Introduction

Sodium-glucose co-transporter 2 inhibitors (SGLT-2i) lower blood glucose concentrations by blocking glucose reabsorption in the kidneys. In addition to their glycemic effect, many large clinical trials have demonstrated reductions in hospitalization for heart failure (HF), cardiovascular death, all-cause mortality, and slowed the progression of chronic kidney disease (CKD) [1]. National guidelines recommend the use of SGLT-2i in patients with type 2 diabetes (T2D), particularly with HF or CKD [2]. New guidelines expanded the role of SGLT2is medications in preserved and reduced heart failure ejection fraction [3]. Based on the results of EMPA-KIDNEY trial, the SGLT2 inhibitor empagliflozin was also FDA approved a new indication for the treatment of adults with chronic kidney disease (CKD) regardless of diabetes diagnosis [4,5]. Despite the robust evidence and guideline recommendations, the prescribing of SGLT-2i remains low in real-world practice [6].

Medication adherence is defined as the “active, voluntary and collaborative involvement of the patient in a mutually acceptable course of behavior to produce a therapeutic result” [7]. Appropriate prescription drug use is a public health challenge. This is specifically a challenge among patients with chronic diseases.

Adherence to medication is a multifaceted topic influenced by a diverse range of patient and system factors. These factors encompass age, gender, socioeconomic status, disease state, pill burden, as well as other systemic considerations like affordability, insurance coverage, and FDA-approved indications [8,9,10].

**Figure 1:**
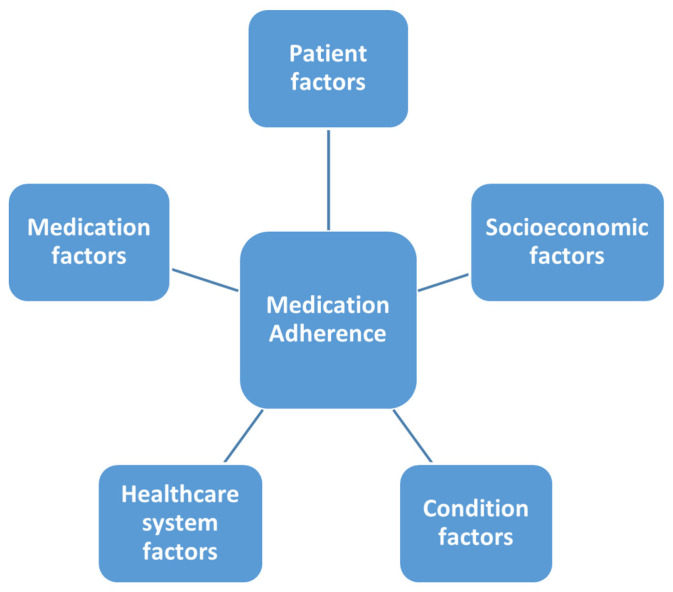
Conceptual model for contributing factors to medication adherence [11].

Many conceptual models have been developed to help understand the impact of the above factors and their contribution to medication adherence. The conceptual framework guiding this research was based on components of the adaptable framework presented by Kai Qi and colleagues [11]. The conceptual figure adopted from the systematic review is presented in Figure 1.

Based on the conceptual model, variables related to patient and condition factors such as age, gender, race, ethnicity are defined as adherence independent variables. Comorbid conditions such asT2D, HF and CKD are also known to play a role in medication adherence.

Another important variable that is listed within the conceptual model is the healthcare system factor. Eaddy et al. demonstrated that an increasing patient share of medication costs was significantly associated with a decrease in adherence [12]. Another study showed that co-insurance changes may lead to decreased adherence to proven effective therapies, particularly for overpriced agents with higher patient cost share [13]. Co-insurance adjustments may disproportionately affect adherence to proven effective disease management. Other barriers to medication adherence include lack of insurance coverage and formulary restrictions [14]. The above studies emphasized the delicate balance between cost considerations and optimal patient care and provided insights on the need to incorporate the financial factors within the variables determining patient acquisition and consequent adherence to chronic medication regimens.

As for the outcome variable in question, adherence to medications have generally been studied as a binary measure (adherent/nonadherent). The use of proportion of days covered metric (PDC) was one of the outcome variables that have been widely used. The cut-off value of PDC was extensively researched [15,16]. This cut-off value was defined as PDC of 0.8–0.9 in most studies which was accompanied by clinical laboratory or physiological measures.

Based on the above independent variables, several studies were conducted to evaluate machine learning in adherence studies. Such studies were conducted to evaluate and predict patient’s adherence patterns and to implement a model to proactively identify patients at higher risk of non-adherence. Zullig, et al. evaluated predictive modeling using statins’ adherence using Medicare part A, B and D claims to evaluate if predictive analytics can proactively determine which patients are at risk of nonadherence, thus allowing for timely engagement in adherence-improving interventions [17]. Another predictive modeling study was conducted by Gu, et al. where the researchers applied various ensemble learning and deep learning models to predict medication adherence among patients’ self-administering injectable medication at home. The prediction model was based on the use of smart sharp disposal bins data to evaluate patient’s adherence to the injectable drug. Thus building an algorithm to identify high risk of non-adherence [18].

As a relatively newer class, SGLT-2i adherence has not been studied extensively, and there is a need for tools that can help to predict adherence patterns in chronic conditions. Our scientific question is whether we can predict SGLT2i adherence in T2D, HF, and CKD patients. Thus, herein, the study’s primary aim is to build a model to predict SGLT-2i adherence in ambulatory care setting using electronic medical records (EMR) in EPIC Datasets along with patient’s prescription filling history. 

The secondary aim is to evaluate diabetes control by comparing glycated hemoglobin between the compliant and non-compliant group defined by proportion of days covered (PDC > 0.8 and <0.8) throughout the study, the definition of compliance cut-off will be reviewed within the methods section. Chronic kidney disease progression was evaluated based on estimated glomerular filtration rate (eGFR) value among both groups. 

## 2. Materials and Methods

### 2.1. Study Design

This is a retrospective observational study, collected data within the timeline between 1 January 2020, and 30 April 2021, of adult patients receiving a prescription for SGLT-2i at UC San Diego Health ambulatory pharmacies.

### 2.2. Participants

Adult patients defined as 18 years and older with a diagnosis of T2D, CKD, or HF (by ICD10 coding) prescribed any SGLT-2i with a minimum of 1 insurance claim within the study period were included. SGLT-2i included: canagliflozin, dapagliflozin, empagliflozin, ertugliflozin, as monotherapy or in a combination drug formulation. Patients with a solid organ transplant or those receiving dialysis were excluded. This study was approved by University of California-San Diego Health Systems institutional review board (210767), and a waiver of consent was approved. 

### 2.3. Data Collection and Outcomes

Data collected included an extensive array of patient-related information such as age, gender, race/ethnicity, diagnosis, comorbidities, medication, copay, laboratory values, and insurance plan payment from the electronic health record (EHR). Duplicate data entries and irrelevant insurance claims were removed. Insurance claims were grouped based on index duration time per patient: 30-day index (0–30 days covered), 60-day index (31–60 days covered), and 90-day index (61–90 days covered). Prescription filling duration was grouped based on the duration of dispensed medication with individualized index date to aggregate three main data times: baseline to 30 days, 60 days and 90+ days of SGLT-2i dispensed medication record. Patients with a new start and who have been using SGLT-2 chronically were included. The rational was based on clinical evidence that adherence trajectory has been linked with the initial 3–4 months of medication filling and the use of the dependent and independent variables within machine learning can predict the importance of each variable across the different data points [19] Baseline laboratory values were captured at the date of prescription filled +/−3 months. Incorporating temporal dimension to the dataset, baseline values above were included to reflect patient’s health status at initiation of therapy. The primary outcome for measuring adherence amongst study subjects was the proportion of days covered (PDC) based on pharmacy insurance claims. The PDC is used to estimate medication adherence by calculating the proportion of days in which a patient has access to the medication, over a given period of interest. PDC was calculated over the study period defined as the period of interest. PDC was calculated manually and cross checked with EPIC autogenerated PDC value for each patient:


*PDC = number of days covered by the pharmac supplied medication/number of days a medication is needed*

PDC was treated as a binomial variable with a cut point of ≥0.8 to divide the cohort into two groups: high (≥0.8) and low (<0.8) adherence groups. The determination of adherence and non-adherence categories based on PDC thresholds of >0.8 and <0.8 were made in accordance with studies published in adherence research [20]. Even though recent data has shown that a higher PDC cut-off value (>0.8) been recommended for a stricter HbA1c target (≤7%), our targeted PDC was set to 0.8 to match chronic conditions adherence values besides T2D.

#### 2.3.1. Statistical Analysis

Descriptive statistics were used to summarize demographic and clinical characteristics of the cohort. Categorical data was summarized using percentages. Continuous data was summarized using the mean with standard deviation or median with interquartile range, depending on the distribution of the data. 

#### 2.3.2. Predictors

The following predictor variables were screened: age, race/ethnicity, gender, comorbidities, glycosylated hemoglobin, glomerular filtration rate, medication, copay assistance amount, amount payer plan paid, and insurance plan type. Welch’s *t*-test was used to compare continuous variables between the two-adherence groups, and a *p*-value of <0.05 was considered statistically significant.

#### 2.3.3. Predictive Model

We examined backward and forward feature selection, and lasso regression, and constructed a Classification and Regression Tree (CART) model. Decision tree methods with k-fold cross-validation (k = 10). To construct our predictive model, we adopted a comprehensive approach that included LASSO (Least Absolute Shrinkage and Selection Operator), CART (Classification and Regression Trees), and both backward and forward feature selection methods to identify the most effective predictive model. The LASSO technique served as a regularization method, assisting in feature selection by penalizing the absolute size of regression coefficients. This helps mitigate overfitting and selects a subset of relevant patient features. On the other hand, the CART model facilitated the generation of decision trees through recursive partitioning, capturing intricate relationships within the data and offering interpretability in clinical settings. We evaluated the model performance using measures such as the Receiver Operating Characteristic/Area Under Curve (ROC/AUC), accuracy, sensitivity, and specificity. Data was split into training and testing, with allocation of 75% for training and the remaining 25% for testing. The partitioning of the dataset into training and testing subsets was accomplished using a randomization approach in R Studio. Specifically, we employed the randomization functions available in R Studio to ensure an unbiased and representative allocation of data for model training and subsequent performance evaluation. Accuracy was calculated for each model using the test data. All analyses were conducted in R Studio (version 2022.07.0).

#### 2.3.4. Software

The study was conducted using R-packages (4.2.1) including MASS (7.3-60.0.1), caTools (1.18.2), stats (3.6.2), ReadXl (1.4.3), GG plot 2 (3.5.0), Caret (6.0-94), GLMNET (4.1-8), Leaps (3.1), ROCR (1.0-11), Desctools (0.99.54), Dplyr (1.1.4), olsrr (0.5.3), and Rpart.plot packages (3.1.2).

#### 2.3.5. Comparative Analysis

To further evaluate the directional relationship among the predictors in relation to the outcome (PDC) a linear regression analysis was conducted in R studio using LM package. The linear regression model was specified with the Proportion of Days Covered (PDC) as the dependent variable and relevant predictors identified in the exploratory analysis. These predictors included demographic variables (e.g., age, gender), clinical factors (e.g., baseline A1c levels), socioeconomic status indicators (e.g., insurance coverage, copayments), and other relevant variables influencing medication adherence.

Model Fitting: The LM package in R Studio was utilized to fit the linear regression model to the data. The lm() function was used to specify the model formula, with the dependent variable PDC regressed on the selected predictors. The lm() function estimates the coefficients for each predictor, indicating the strength and direction of their relationship with the outcome variable.

Assessment of Model Fit: The adequacy of the linear regression model was assessed using diagnostic measures such as R-squared (R^2^) and adjusted R-squared (adjusted R^2^).

Interpretation of Results: The coefficients estimated by the linear regression model provide insights into the direction and magnitude of the relationship between each predictor and medication adherence (PDC). Positive coefficients indicate a positive relationship, while negative coefficients suggest a negative relationship. The significance of each predictor was assessed based on *p*-values, with lower *p*-values indicating stronger evidence against the null hypothesis of no effect.

Random effect was employed in the analysis to help mitigate the potential bias introduced by the inherent correlation between observations within each patient.

## 3. Results

### 3.1. Cohort Characteristics

**Figure 2:**
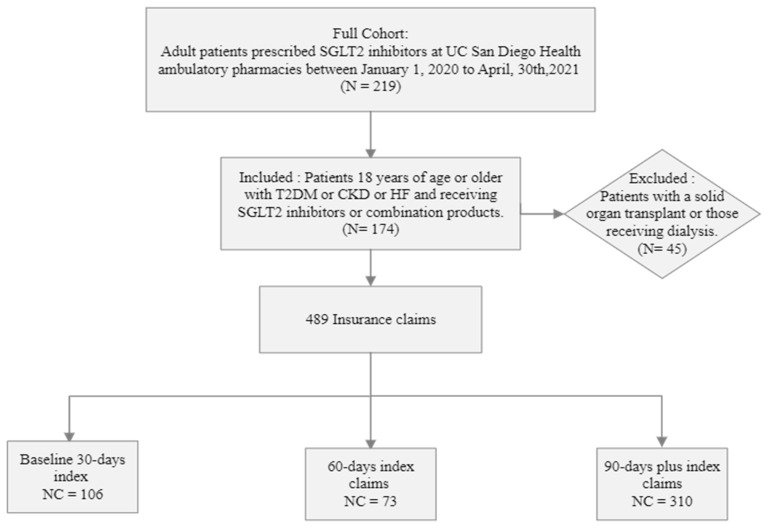
Flow chart illustration of patient cohort for the retrospective study design. *n* = number of patients, NC = number of insurance claims, Baseline 30-, 60-, 90-days index is defined as grouping of claims provided for an average of 30, 60, 90-days and beyond average duration.

A total of 174 patients with 489 insurance claims were included in the analysis. One hundred and six claims were within the first 30 days, 73 in 60 days, and 310 in 90 days fills (Figure 2). The demographic and clinical characteristics of the patient cohort are summarized in Table 1. The median age was 58 years (IQR), and a higher dominance of the male gender was observed. A vast majority of patients taking SGLT-2i had diabetes (83.6%) and the lowest representation of patients taking SGLT-2i was patients with heart failure and CKD. In the total cohort, the baseline HbA1c was 8% and the baseline eGFR was 54 mL/min/1.73 m^2^.

Using a PDC threshold of 0.8 for adherence, 88 (51%) were considered adherent. The adherent group had a lower eGFR (50.3 vs. 57 mL/min/1.73 m^2^, *p* < 0.001) compared to the non-adherent group.

HbA1c values from baseline till 90 days post-treatment duration were consistently higher in the non-compliant group: 7.4% vs. 9.6%, *p* < 0.001 for the PDC ≥ 0.80 and PDC < 0.80, respectively.

Baseline eGFR was 55.18 mL/min/1.73 m^2^ vs. 54.23 mL/min/m^2^ at 90 days. The mean eGFR at the end of the study (minimum of 90 days of treatment) was statistically different between the groups: 53.1 vs. 59.6 mL/min/1.73 m^2^, *p* < 0.001 for the PDC ≥ 0.80 and PDC < 0.80, respectively.

Eighty-seven percent of patients were commercially insured. Assistance programs’ use (such as manufacturer coupons and health system patient assistance programs) didn’t exceed 2% of the total cohort. There was a higher representation of private vs. federal insurance claims within this suburban community. A mean copay (patient responsibility to pay) was $9.76, and the insurance plan paid a mean of $509 per insurance claim.

It’s worth noting that the percentage of patients with federally funded insurance differs between the two groups based on their medication adherence. Among patients with PDC ≥ 0.8, only 4% have federally funded insurance, while among patients with PDC < 0.8, the percentage increases to 12%.

The Adherent group had a mean copay of $12.56 vs. $5.07 for the non-adherent group. As for insurance payment, the adherent group had a mean of $547.30 vs. 430.23 for the non-adherent group. The adherent group had an average high assistant pay vs. non adherent at $4.97 vs. $2.81 which was non-significant.

As for insurance plans, the adherent group had a higher representation of commercial insurance (303 vs. 125 claims for the non-adherent group). With similar representation of federally funded insurance claims among adherent and non-adherent groups (14 vs. 18, respectively). Please refer to Table 2 for detailed information about SGLT-2i insurance claims.

### 3.2. Predictive Modeling

#### Feature Selection

Best variables were selected with a significant *p* value < 0.05. The selection was based on the lowest Akaike information criterion (AIC). In addition, we calculated the C(*p*), RMSE, and rsquare. Forward selection model results in selecting a total of 8 variables based on different metrics. The backward selection model resulted in selecting a total of 18 variables excluding 5 variables based on the same metrics above.

To select among the models above we calculated based on area under the curve (AUC) in Receiver operating characteristic curve (ROC).

Among all the tested predictive models, classification and regression tree (CART) model had the highest accuracy and area under the curve (AUC) compared to backward, forward, and lasso predictive models (AUC = 74%, accuracy = 82%).

**Figure 3:**
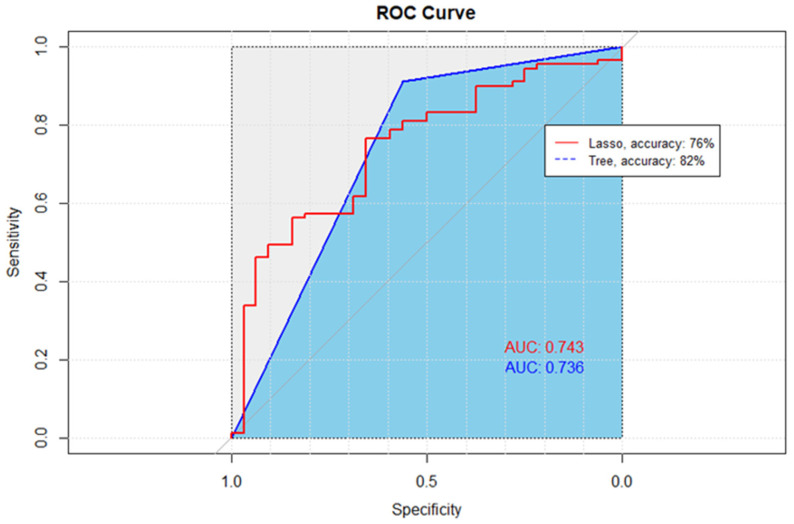
Receiver Operating Characteristic (ROC) curves comparing the performance of Lasso and CART methods. The diagonal line, representing the performance of a random classifier, serves as a baseline for comparison. The ROC curve for the Lasso method, denoted by the red line, exhibits an accuracy of 76%, while the ROC curve for the CART method, depicted in blue, achieves a higher accuracy of 82%. The ROC curves illustrate the trade-off between the True Positive Rate (sensitivity) and the False Positive Rate, with curves further away from the diagonal indicating superior performance.

The Lasso and CART model both provided a close AUC value (74%) (Table 3). Lasso had a higher sensitivity score of 94%. However, since accurately identifying non-adherent patients and overall prediction accuracy are more important, the CART model’s higher specificity and accuracy outperforms the Lasso model (Figure 3).

CART analysis resulted in 21 variables included within the final model and an AUC of 74% (Figure 3). Based on the final model, glycated hemoglobin concentration was one of the most important predictors. An inverse relationship exists between baseline HbA1c value and adherence as measured by PDC. The final model’s accuracy, specificity, and sensitivity were 82%, 69%, and 85%, respectively.

**Figure 4:**
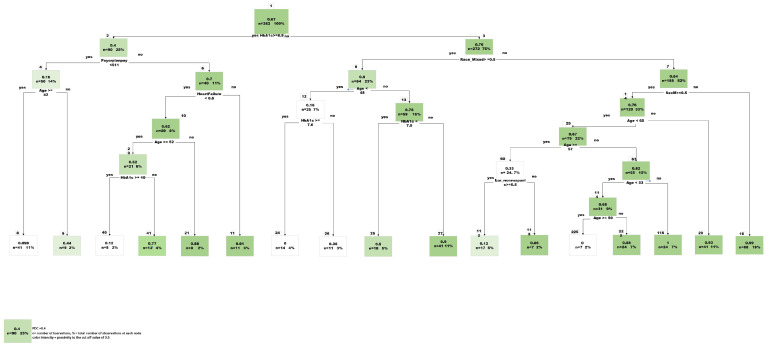
Final Predictive Tree model, CART (Classification and Regression Tree).

To further evaluate the directional relationship among the predictors in relation to the outcome (PDC) a linear regression analysis was conducted in R studio using LM package. The resulted analysis confirmed the relevance of each predictor on PDC illustrated in (Appendix A: Table A1). It is important to note that HbA1c shows a strong negative correlation with PDC. The higher initial HbA1c has a very strong correlation for a lower compliance rate. This is also confirmed with the decision tree where HbA1c value of 8.9 is a deciding node to different routes of compliance scores (Figure 4).

Another important predictor to note is the amount paid by the plan payor. There was a positive correlation of higher plan payment with a better compliance (statistically significant).

Male gender was negatively correlated with PDC. Being a male puts the patient into a lower compliance group. There was a positive correlation of adherence in relationship to specific SGLT2is agents as empagliflozin/metformin and canagliflozin with a significant *p* value < 0.05.

## 4. Discussion

This study examined predictors of SGLT-2i adherence in patients by analyzing pharmaceutical insurance claims derived from an electronic health record dataset. Predictive modeling can be a crucial method to help improve patient care and provide a proactive approach to resolve any potential adherence issues and its consequent complications. The utilization of insurance claims offers an opportunity to investigate the additional financial aspect and it’s impact on patient’s adherence patterns. Such a proactive approach has been the key to improving patient overall health and has positive financial impact that is worth further investigation and implementation [21].

This study investigated several key predictors of SGLT-2i adherence, some variables played an important role in building the predictive model. Notably, HbA1c, age, plan payment amount, race/ethnicity, and gender emerged as important predictors of adherence. 

A major variable in the model was HbA1c value. HbA1c is a critical marker of glycemic control, and it appeared in the model as a significant predictor of patient’s adherence. Similarly, Wu et al. found that the last HbA1c value, age, and cost of hypoglycemic drugs, were important predictors among 16 predictors of adherence to diabetes treatment [22]. The HbA1c value can be used to identify patients at risk for lower adherence, empowering the pharmacist to assign more intensive follow-up and comprehensive medication management. Nichols et al. showed that the average decrease in HbA1c concentrations was 0.6% vs. 0.4% in newly diagnosed patients with diabetes who had a PDC ≥ 0.80 and PDC < 0.80, respectively [23]. This emphasizes the tangible clinical benefits associated with robust medication adherence in context of glycemic control [24].

The predictive model showed a correlation between the insurance payment amount and adherence where the higher percentage the insurance paid was associated with a higher adherence rate. The share of cost and adherence patterns were investigated by Aziz, et al. in a systematic review [25]. The interesting finding however was that although medication adherence was improved with the reduction of cost-sharing such as lower copayment, higher drug coverage, and prescription cap, patients with full-medication subsidies payment scheme (received medication at no cost) were also found to have poor adherence to their medication. Cost sharing, insurance formulary tiers and patient assistance programs may need to be further investigated as barriers or facilitators of medication acquisitions and subsequent adherence implications [26].

Another variable that the decision tree identified was age. Age was presented as a decision tree node in multiple nodes and was related to medication adherence predictions. Specifically, the lower age group exhibited a higher predictive Proportion of Days Covered (PDC) value, indicating better adherence. However, the relationship between age and adherence has yielded mixed results in various studies. For instance, a retrospective study by Habib et al. found a strong correlation between higher age, higher socioeconomic status, and improved adherence [27].

One interesting finding is the lower 90 days post-treatment mean eGFR rate in the compliant group vs. non-compliant. This could be related to the retrospective nature of the study. Another explanation could be related to the initial eGFR SGL-T2i “dip” where initially, the eGFR decreases as part of the long-term nephroprotective mechanism. Kidney protection has been proven in several randomized controlled trials [28,29,30,31]. Their preservation of kidney function is thought to be mainly mediated through the reduction in glomerular hypertension mediated through tubule-glomerular feedback. Due to the small sample size, missing data in eGFR lab values, and the lack in adjustment for comorbidities; the results may not depict the full picture of kidney protection effect.

Overall, the study has some limitations that are worth stating. One of the study design choices was the use of proportion of days covered (PDC). We choose proportion of days covered (PDC) as a binary outcome since this is a clinically relevant outcome in clinical practice. Specifically, a PDC cutoff of equal or more than 0.8 is defined as adherent for medications in clinical practice. Nevertheless, PDC has its own inherent limitations. PDC may fail to explain certain treatment gaps. For example, PDC may not explain a treatment holiday, patient taking samples or receiving medications from a different pharmacy. There is not a second validation method to account for such scenarios with PDC alone.

The study sample size is small and is based solely at the ambulatory pharmacies from a single institution. This scope may limit the generalizability of findings, which may not reflect all the commercially available insurances in different geographical areas, different race/ethnicity groups, or socio-economic status. The monocentric nature of our study may challenge the extrapolation of results to broader and more heterogeneous patient populations.

The retrospective study design creates limitations. Historical data introduces certain limitations that can be described by the standard of care measurement bias, loss to follow-up and missing data. 

The data collection study period may impose a temporal limitation. Since the data collection period ran from 1 January 2020, till 30 April 2021, this data may not fail to reflect the most current adherence patterns and predictors. An important limitation pertains to the relatively short follow-up measurement period. Longer follow-up periods could provide a more comprehensive understanding of adherence behaviors over time. Such adherence patterns evolve over time and are influenced by various factors. Future research with a longer follow-up period would contribute to a better understanding of medication adherence.

The observational and retrospective nature of the study was able to establish correlation but not causation. Unmeasured confounding variables may influence the results. Thus, future research should collect prospective data and analyze the impact of such variables on adherence patterns to improve external validation of the predictive model.

It is worth noting that the study collection period happened to occur within COVID-19 pandemic. It is plausible that this could have impacted medication adherence patterns. Factors as changes to patients’ routines and economic challenges may have an impact. The reason for non-adherence was hard to investigate in a retrospective manner and as such it was challenging to evaluate the unique circumstances imposed by the global health crisis. This pandemic may have positively or negatively impacted the adherence patterns. All pharmacies included in this study offered free delivery of medications and an assistance program to overcome financial burdens.

It is important to existing literature on medication adherence had implemented several strategies to mitigate low adherence including but not limited to technology-based interventions (as electronic pill organizers, smartphone applications, etc., …), addressing socioeconomic barriers and enhancing patient-provider communication. Such tools can be used proactively to implement an early prevention plan to boost adherence.

A future study can evaluate the impact of race, health education, access, and health disparities among communities in regard to medication adherence. Conducting a multi-national study from different institutions may help increase the generalizability of the predictive adherence model.

A prospective study design may also help collect enough data points and resolve the issue of missing data that we faced in the retrospective design.

## 5. Conclusions

The utilization of sodium-glucose co-transporter 2 inhibitors has emerged as promising therapeutic agents for patients with type 2 diabetes, heart failure and chronic kidney disease, offering glycemic control and cardiovascular benefits.

This retrospective study, conducted at UC San Diego Health ambulatory pharmacies, aimed to predicted SGLT-2i adherence using electronic medical records and demographic variables. While this study provided insights regarding adherence patterns, it is crucial to consider its implications for clinical practice and future research.

HbA1c, age, gender, and payor plan payments are important predictors of medication adherence for diabetes care. Using these variables, the community pharmacist can identify at-risk patients and design comprehensive medication management programs to improve adherence and diabetes outcomes. Higher adherence will reduce comorbidities, decrease hospitalizations, and reduce overall healthcare costs, specifically in chronic conditions, including diabetes mellitus, heart failure, and kidney failure [32]. Thus, a predictive analytics approach could be used to demonstrate how event-based data can form the basis for identifying patients who are at risk for future non-adherence and, consequently, more complications [33].

Improving medication adherence remains a critical goal in optimizing the care of patients with chronic conditions. This study represents a step toward improving that goal.

The trajectory of future research is to elaborate and identify at risk of non-adherence patients’ variables to prevent complications. Beyond the immediate clinical implications, the broader impact of enhanced adherence, predictive modeling can be implemented to improve personalized preventative care.

## Figures and Tables

**Table 1:** Cohort characteristics in adherent and non-adherent patient groups.

Variable	Total Cohort (*n* = 174)	PDC ≥ 0.8 (*n* = 88)	PDC < 0.8 (*n* = 86)	*p*-Value
Age, median (IQR)	58 (51–66)	59 (48–68)	58 (52–65)	0.78
Sex, *n* (%)				
Female	39 (22.4%)	24 (27.3%)	15 (17.4%)	0.12
Male	135 (77.6%)	64 (72.7%)	71 (82.6%)	
Ethnicity, *n* (%)				
Hispanic	55 (31.6%)	24 (27%)	31 (36%)	0.21
Non-Hispanic	119 (68.3%)	64 (73%)	55 (64%)	
Race, *n* (%)				
White	74 (42.5%)	42 (47.7%)	32 (37.2%)	0.099
African American	20 (11.5%)	13 (14.7%)	7 (8.1%)	
American Indian	2 (1.1%)	0 (0%)	2 (2.3%)	
Asian	13 (7.5%)	4 (4.5%)	9 (10.5%)	
Mixed	65 (37.4%)	29 (32.9%)	36 (41.8%)	
Prescribing Indication, *n* (%)				
Diabetes Mellitus	151 (86.8%)	69 (78.4%)	82 (95.3%)	0.12
Heart Failure	66 (37.9%)	40 (4.5%)	26 (30.2%)	
Kidney Disease	21 (12.1%)	10 (11.4%)	11 (12.5%)	
Baseline HbA1c (SD)	8.04 (2.39)	7.1 (1.5)	8.98 (2.3)	<0.001
Baseline eGFR (SD)	53.6 (7.4)	50.3 (11.1)	57 (6.4)	<0.001

**Table 2:** SGLT-2i Insurance claims and payments.

Medication/Insurance Plan Type	Total Claims (*n* = 489)	PDC ≥ 0.8 (*n* = 338)	PDC < 0.8 (*n* = 151)	*p* Value
Dapagliflozin, *n* (%)	107 (21.9%)	82 (24%)	25 (17%)	<0.0001
Canagliflozin, *n* (%)	22 (4.5%)	22 (7%)	0 (0%)	
Empagliflozin, *n* (%)	349 (71.3%)	229 (68%)	120 (79%)	
Empagliflozin/metformin, *n* (%)	5 (1%)	5 (1%)	0 (0%)	
Ertugliflozin, *n* (%)	6 (1.2%)	0 (0%)	6 (4%)	
Insurance Plan Type, *n* (%)				
Commercial, *n* (%)	428 (87.5%)	303 (90%)	125 (83%)	<0.001
Commercial with Assistance, *n* (%)	20 (4.1%)	16 (5%)	4 (3%)	
Federally Funded, *n* (%)	32 (6.5%)	14 (4%)	18 (12%)	
Federally Funded with Assistance, *n* (%)	3 (0.6%)	3 (1%)	0 (0%)	
Assistance Program, *n* (%)	6 (1.2%)	2 (1%)	4 (3%)	
Patient Copay, mean (SD)	$9.76 (26.17)	$12.27 ($30.31)	$4.15 ($10.81)	<0.001
Payor Plan Pay, mean (SD)	$509.22 (282.45)	$547.3 ($305.38)	$423.99 ($197.99)	<0.001
Assistance Pay, mean (SD)	$3.92 (16.45)	$4.97 ($18.18)	$1.6 ($11.34)	0.255

**Table 3:** Summary of Lasso and CART model measures.

Predictive Model	Accuracy	Sensitivity	Specificity	AUC
Lasso	76%	94%	25%	74%
CART	82%	85%	69%	74%

## Data Availability

The data presented in this study are available on request from the corresponding author. The data are not publicly available due to privacy restrictions.

## Appendix A

**Table A1:** Linear regression analysis: Final model predictors vs. PDC outcome.

	Estimate	Std. Error	*t* Value	Pr (>|t|)
(Intercept)	9.04 × 10^−1^	4.06 × 10^−1^	2.228	0.026353
Age	2.73 × 10^−3^	2.07 × 10^−3^	1.319	0.187991
HbA1c	−4.96 × 10^−2^	1.09 × 10^−2^	−4.548	6.97 × 10^−6^
PayorPlanPay	3.04 × 10^−4^	8.01 × 10^−5^	3.79	0.000171
SexM	−1.04 × 10^−1^	4.73 × 10^−2^	−2.205	0.027934
Race_Asian	2.87 × 10^−1^	3.01 × 10^−1^	0.954	0.340608
Race_White	1.38 × 10^−1^	2.97 × 10^−1^	0.466	0.641451
Federal	−3.32 × 10^−1^	2.56 × 10^−1^	−1.299	0.194441
Race_Mixed	8.23 × 10^−2^	3.02 × 10^−1^	0.273	0.785327
Commercial	−3.35 × 10^−1^	2.42 × 10^−1^	−1.385	0.166883
Eth_NonHispanic	1.08 × 10^−1^	6.96 × 10^−2^	1.552	0.121452
Race_AfricanAmerican	1.89 × 10^−1^	2.98 × 10^−1^	0.634	0.526556
Diabetes	−5.76 × 10^−2^	6.94 × 10^−2^	−0.831	0.4066
Empagliflozin	8.02 × 10^−2^	5.85 × 10^−2^	1.371	0.171036
Ertugliflozin	−8.56 × 10^−1^	1.95 × 10^−1^	−4.386	1.43 × 10^−5^
AssistancePay	2.56 × 10^−3^	1.36 × 10^−3^	1.877	0.061142
PatPay	1.96 × 10^−3^	8.46 × 10^−4^	2.319	0.020844
Assistance_Prog	−4.48 × 10^−1^	2.99 × 10^−1^	−1.496	0.135427
Empagliflozin/metformin	9.33 × 10^−1^	2.58 × 10^−1^	3.621	0.000326
Canagliflozin	4.59 × 10^−1^	1.15 × 10^−1^	3.985	7.85 × 10^−5^
Commercial_Assis	−1.88 × 10^−1^	2.67 × 10^−1^	−0.702	0.482787
